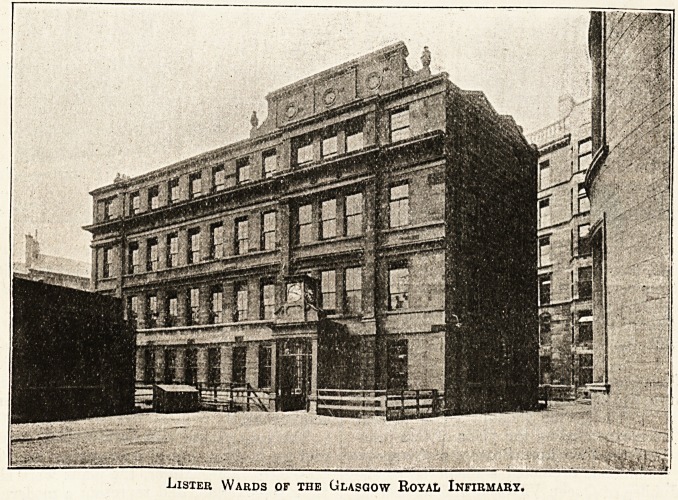# The Royal Infirmary, Glasgow: The Genesis of the Scheme

**Published:** 1914-11-14

**Authors:** 


### THE ROYAL INFIRMARY, GLASGOW.

### I. The Genesis of the Scheme.

His Majesty the King, after opening the Royal Infirmary, which had been rebuilt throughout, declared it to be a fine hospital, and that description is likely to remain a proud fact for the citizens of Glasgow, providing the administration throughout this vast establishment is entrusted to a really able and knowledgeable administrator. Some idea of the size of this great hospital may be gathered from the following figures, which were supplied to the Press: The total floorage exceeds 500,000 sq. ft., or nearly twelve acres; fifty-two miles of branch electrical cables have been used, and over ten and a half miles of power and main cables. The cubic content of the entire building is 12,000,000 cubic ft., which constitutes it by far the largest building in the city of Glasgow. It contains 5,000 electric lights, fifty electric motors, and seventy-five private telephones. The number of persons supplied from the hospital kitchen on the day after the opening exceeded 1,100. When fully equipped, the hospital will contain accommodation for 700 patients.

The Medical Block Front (facing Cathedral Square).

Few, if any, of our modern hospitals in this country have had the advantage of an amount of careful and knowledgeable thought equal to that which has been expended on the plans of this infirmary. In 1897 the then Lord Provost of Glasgow, Sir David Richmond, promoted a memorial of the Diamond Jubilee of Queen Victoria, and formed an executive committee of prominent citizens, appointed at a public meeting, to raise £100,000. This Executive Committee ultimately enlarged their scheme by obtaining plans for the reconstruction of all the ward blocks, and finally adopted and submitted to the managers a set of plans, which had been prepared by Mr. James Miller, F.R.I.B.A., architect, Glasgow. The managers felt, however, that much more was necessary to complete and efficiently equip a great infirmary. They therefore elaborated a complete scheme, involving a total expenditure of £500,000, which will probably amount in the end to £600,000. It was originally intended to provide accommodation for 660 patients, but the number will be increased to 700 patients when the whole establishment has been brought into full working order. The managers therefore issued the following statement: "After long and anxious consideration by the managers and staff, the plans—*i.e.* Mr. James Miller's—were remitted for revision, in so far as the internal arrangements were concerned, to Sir Henry Burdett, K.C.B., and Dr. D. J. Mackintosh, M.V.O., both acknowledged experts, to examine and make suggestions as they may think advisable for their improvement in detail or as a working whole, bearing in mind that the general ground plan of the main ward blocks must not be departed from and that the number of beds must not be diminished."

The experts had therefore a grave responsibility cast upon them, but in co-operation with the architect, Mr. James Miller, the plans were adjusted to their satisfaction, and represent a modern hospital, up to date, containing several novel features of special interest. The old infirmary exhibited throughout the great defect of an absence of any isolation for each ward, or block of wards, and failed to secure the fullest hygienic conditions in regard to the sanitary blocks, the theatres, and other departments where isolation and the strictest attention to light and air are so essential to the efficiency of the ward unit.

It will be seen from the illustrations which we publish that Mr. Miller has succeeded in giving an attractiveness and importance to the exterior of this great hospital which does him infinite credit, and is in marked contrast to the work of some modern architects responsible for hospital buildings. It is quite certain that when the cost of a new, modern, up-to-date hospital in this country amounts to anything like £700 per bed there should be ample money to render a really competent and great architect full opportunity to give to the exterior of the hospital buildings an attractive force, which cannot fail to help the managers materially in raising money for its maintenance and in popularising the institution with the great mass of the people to whose needs when ill it will mainly contribute. Cheerfulness in the sick is the high road to recovery. Attractiveness of design for a great hospital building need not mean excessive expenditure. An attractive elevation is undoubtedly a factor which has not only an æsthetic and artistic value, but may be made of material importance financially and hygienically, for the reasons we have just given.

General View from the East.

Lister Wards of the Glasgow Royal Infirmary.

**[We shall publish the plans, with a full description, in our next issue.]**

## Figures and Tables

**Figure f1:**
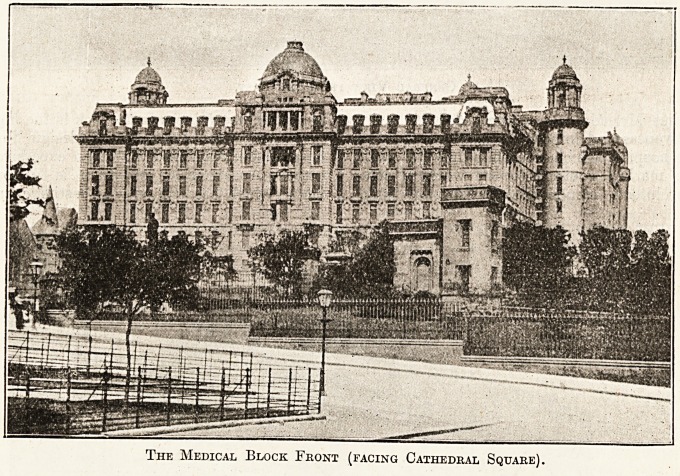


**Figure f2:**
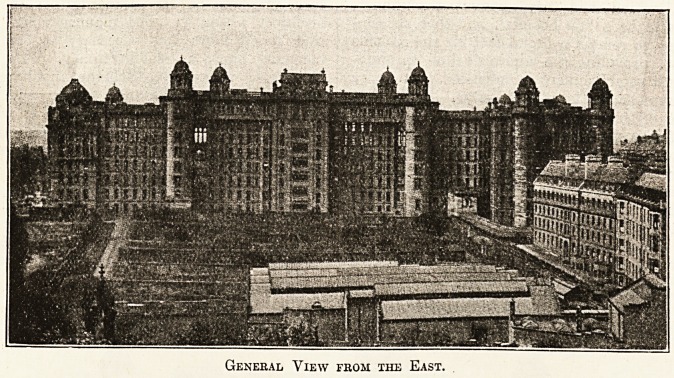


**Figure f3:**